# Hybrid Molecules of Benzylguanidine and the Alkylating Group of Melphalan: Synthesis and Effects on Neuroblastoma Cells

**DOI:** 10.3390/jcm12134469

**Published:** 2023-07-03

**Authors:** Gernot Bruchelt, Chihab Klose, Matthias Lischka, Marietta Brandes, Rupert Handgretinger, Reinhard Brueckner

**Affiliations:** 1 Children’s University Hospital, Hoppe-Seyler-Str. 1, D-72076 Tuebingen, Germanyrupert.handgretinger@med.uni-tuebingen.de (R.H.); 2 Institute of Organic Chemistry, Albert-Ludwigs-University, Albertstr. 21, D-79104 Freiburg, Germany; matthiaslischka@gmx.de

**Keywords:** neuroblastoma, melphalan (M), (*meta*-iodobenzyl)guanidine (mIBG), melphalan benzylguanidine hybrids (MBGs), noradrenaline transporter (NAT), organic cation transporter (OCT)

## Abstract

The therapy of neuroblastoma relies, amongst other things, on administering chemotherapeutics and radioactive compounds, e.g., the (*meta*-iodobenzyl)guanidine [^131^I]mIBG. For special applications (conditioning before stem cell transplantation), busulfan and melphalan (M) proved to be effective. However, both drugs are not used for normal chemotherapy in neuroblastoma because of their side effects. The alkylating drug melphalan contains a (Cl-CH_2_-CH_2_-)_2_N- group in the *para*-position of the phenyl moiety of the essential amino acid phenylalanine (Phe) and can, therefore, be taken up by virtually all kinds of cells by amino acid transporters. In contrast, mIBG isotopologs are taken up more selectively by neuroblastoma cells via the noradrenaline transporter (NAT). The present study aimed at synthesising and studying hybrid molecules of benzylguanidine (BG) and the alkylating motif of M. Such hybrids should combine the preferential uptake of BGs into neuroblastoma cells with the cytotoxicity of M. Besides the hybrid of BG with the dialkylating group (Cl-CH_2_-CH_2_-)_2_N- bound in the *para*-position as in M (pMBG), we also synthesised mMBG, which is BG *meta*-substituted by a (Cl-CH_2_-CH_2_-)_2_N- group. Furthermore, two monoalkylating hybrid molecules were synthesised: the BG *para*-substituted by a (Cl-CH_2_-CH_2_-)NH- group (pM*BG) and the BG *meta*-substituted by a (Cl-CH_2_-CH_2_-)NH- group (mM*BG). The effects of the four new compounds were studied with human neuroblastoma cell lines (SK-N-SH, Kelly, and LS) with regard to uptake, viability, and proliferation by standard test systems. The dialkylating hybrid molecules pMBG and mMBG were at least as effective as M, whereas the monoalkylating hybrid molecules pM*BG and mM*BG were more effective than M. Considering the preferred uptake via the noradrenaline transporter by neuroblastoma cells, we conclude that they might be well suited for therapy.

## 1. Introduction

**Figure 1:**
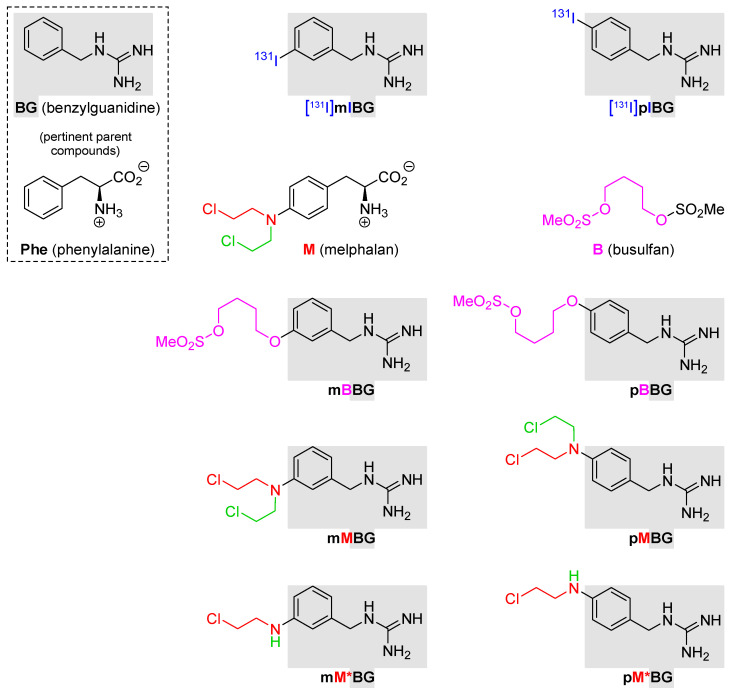
Structural formulas of the compounds described in this study. Line 1: benzylguanidine (**BG**) and the radiotherapeuticals [^131^I]**mIBG** and [^131^I]**pIBG**. Line 2: phenylalanine (**Phe**; essential amino acid); melphalan (**M**), and busulfan (**B**). Line 3: **mBBG** and **pBBG**. Line 4: hybrids with dialkylating entities copied from **M** (**mMBG** and **pMBG**). Line 5: hybrids with monoalkylating entities inspired by **M** (mM*BG and pM*BG). **M**: 4-[*N*,*N*-Bis(2-chloroethyl)amino]phenylalanine; **mMBG**: {3-[*N*,*N*-Bis(2-chloroethyl)amino]-benzyl}guanidine; **pMBG**: {4-[*N*,*N*-Bis(2-chloroethyl)amino]-benzyl}guanidine; mM*BG: {3-[*N*-(2-Chloroethyl)amino]benzyl}-guanidine; pM*BG: {4-[*N*-(2-Chloroethyl)amino]benzyl}-guanidine; **B**: 5-[(Methanesulfonyl)oxy]pentyl methanesulfonate; **mBBG**: 5-[3-(Guanidinomethyl)phenoxy]-pentyl methanesulfonate; **pBBG**: 5-[4-(Guanidinomethyl)phenoxy]pentyl methanesulfonate.

Neuroblastoma is a malignant tumour of the sympathetic nervous system in childhood [1] with a poor prognosis in stage IV patients [2]. Characteristically, most neuroblastoma cells are able to synthesise catecholamines (dopamine, noradrenaline) [3] and they can express the noradrenaline transporter (NAT) for the reuptake of released dopamine and noradrenaline [4]. Besides catecholamines, a few other compounds can be taken up by the NAT, among them (*meta*-iodobenzyl)guanidine (mIBG) [5]. From the 1980s until now [6], radio-iodine-labelled mIBG {first [^131^I]mIBG [7,8], (Figure 1, line 1), soon replaced by [^123^I]mIBG}, has been used for scintigraphic diagnosis [9], and [^131^I]mIBG for therapy [10] of neuroblastoma. It is noteworthy that the benzylguanidine (BG) with ^131^iodine bound in the *meta*-position {[^131^I]mIBG, Figure 1, line 1)} is taken up by NAT-expressing cells equally well as its *para*-isomer [^131^I]pIBG. However, in clinical praxis, the *meta*-substituted forms {[^131^I]mIBG)/[^123^I]mIBG} are used exclusively because of the presence of deiodinases in humans and animals; they remove iodine preferentially from the *para*- and not as readily from the *meta*-position (lower thyroid radioactivity concentration due to lower liberated iodine using the *meta*-substituted forms) [11,12,13].

For the treatment of high-risk neuroblastoma before a hematopoietic stem cell transplantation, the alkylating drugs melphalan (**M**) [14] and busulfan (**B**; traded as “myleran” (GlaxoSmithKline, Stevenage, UK) or “busulfex IV” (Otsuka America Pharmaceutical, Inc, Rockville, MD, USA) [15] are used for conditioning; (Figure 1, line 2). Investigations by the Society for Pediatric Oncology (SIOP; HR-NBL1/SIOPEN) showed that, in such cases, the use of a combination of **M** and **B** is superior to the formerly used protocol using a combination of **M**, carboplatin, and etoposide [16]. Despite the high effectivity of **M** and **B**, they are not included in chemotherapy protocols for neuroblastoma without subsequent stem cell rescue because of their severe toxicities. The unspecific toxicity of **M** is not surprising since the carrier molecule of the dialkylating group is phenylalanine (**Phe**) (Figure 1, line 2), an essential aromatic amino acid, which can be taken up by almost every cell via amino acid transporters [17,18]. In contrast, the uptake of **mIBG** and related compounds in neuroblastoma cells is essentially restricted via the noradrenaline transporter. In some other cells, catecholamines and **mIBG** can be taken up by organic cationic transporters (OCTs) [19,20].

In **M**, the alkylating entity is bound to the *para*-position of the aromatic ring of **Phe**. In order to use the potential of **M** for neuroblastoma treatment in “conventional” chemotherapy, we synthesised and studied hybrid molecules of **BG** and **M**, combining the highly effective alkylating group(s) of the alkylating entity of **M** with **BG** (rather than with **Phe**), with the hope of specific uptake into neuroblastoma cells via the NAT. Conceptually related hybrids between **BG** und **B** (namely, **mBBG** and **pBBG**; Figure 1, line 3) were already synthesised and studied by our groups [21,22].

Besides the hybrid of **BG** with the dialkylating group (Cl-CH_2_-CH_2_-)_2_N- bound in the *para*-position as in **M** (**pMBG**; Figure 1, line 4), we also synthesised a hybrid of **BG**, which is *meta*-substituted by the same (Cl-CH_2_-CH_2_-)_2_N- group (**mMBG**; Figure 1, line 4). Furthermore, two hybrid molecules with monoalkylating (Cl-CH_2_-CH_2_-)NH- groups were synthesised: the *meta*-substituted **BG** (mM*BG; Figure 1, line 5) and the *para*-substituted **BG** (pM*BG; Figure 1, line 5).

The aim of our study was (a) to synthesise the conceptualised four hybrid molecules and (b) to analyse their uptake via the noradrenalin transporter in vitro and their cytotoxic effects.

## 2. Materials and Methods

### 2.1. Synthesis of the Hybrid Compounds mMBG, pMBG, mM*BG, and pM*BG

**Scheme 1:**
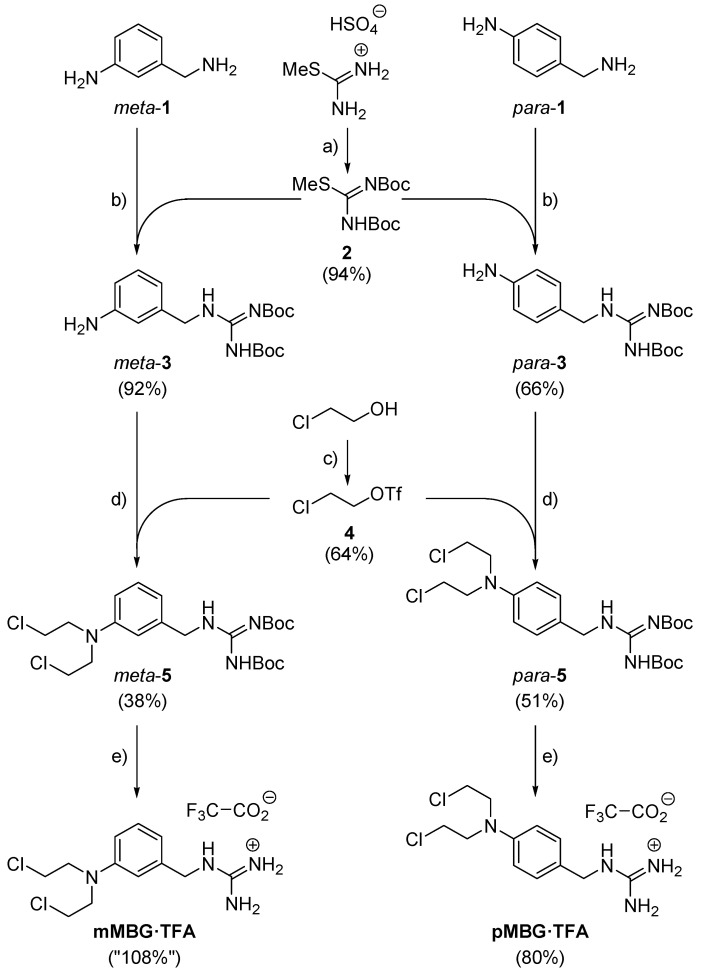
Syntheses of our two MBG hybrid molecules. Reagents and conditions: (**a**) Boc_2_O (3.1 eq.), aq. NaHCO_3_ (xs.), room temp., 5 d; 94% (ref. [26]: 100%). (**b**) 2 (1.2 eq.), NEt_3_ (4.2 eq.), CH_2_Cl_2_, reflux, 20 h for para-4, 6 h for meta-4. (**c**) Tf_2_O (1.1 eq.), pyridine (1.2 eq.), CH_2_Cl_2_, 0 °C, 50 min; 64%. (**d**) 4 (4.0 eq.), K_2_CO_3_ (2.2 eq.), CH_2_Cl_2_, reflux, 18 h. (**e**) F_3_CCOOH/CH_2_Cl_2_ (1:1), room temp., 30 min.

Our syntheses of the **MBG** hybrids **mMBG** and **pMBG**—which we isolated as the corresponding trifluoroacetates **mMBG**·TFA and **pMBG**·TFA—proceeded as shown in Scheme 1. The starting materials were the diamines *meta*- and *para*-**1**, which are commercially available. The higher nucleophilicity of their sp^3^- rather than sp^2^-bound NH_2_ groups allowed regioselective amidinylations of the former by the *N*,*N*’-di-Boc-protected isothiourea **2**, a reagent obtained from *S*-methylisothiourea hydrogensulfate, as described in [23]. The *N*,*N*’-di-Boc-protected amino-**BG**s *meta*-**3** and *para*-**3** resulted in yields of 92% and 66%, respectively [24]. The aniline moiety of these compounds underwent double alkylations with 2-chloroethyl triflate (**4**), which had been prepared from chloroethanol by a known triflation [25]. These alkylations provided the (Cl-CH_2_-CH_2_-)_2_N-containing compounds *meta*-**5** (38% yield) and *para*-**5** (51% yield [24]). The terminating step of the syntheses shown in Scheme 1 was the removal of the two Boc groups. It occurred when we stirred solutions of the respective isomers of compound **5** in trifluoroacetic acid (TFA)/CH_2_Cl_2_ (1:1, *v*:*v*). Evaporation of all volatiles afforded the (Cl-CH_2_-CH_2_-)_2_N-containing benzylguanidine trifluoroacetates **mMBG**·TFA and **pMBG**·TFA. Their syntheses comprised 3 steps in the longest linear sequence and succeeded in total yields of 38% and 27%, respectively.

**Scheme 2:**
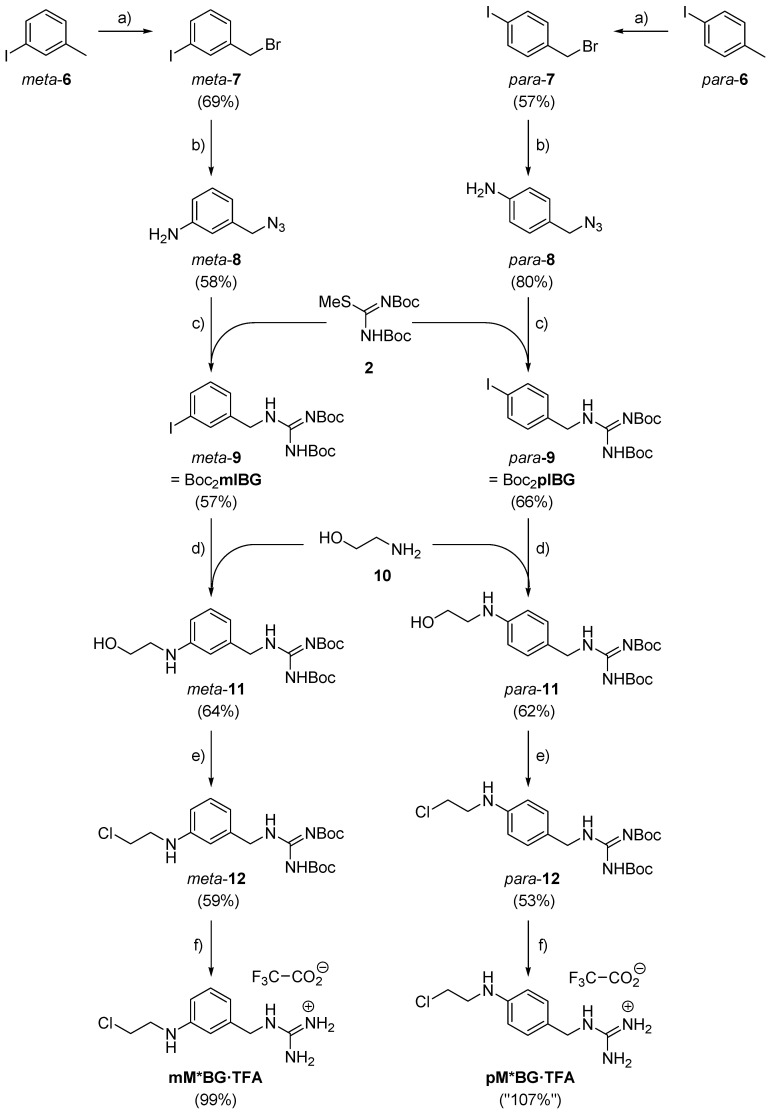
Syntheses of our two M*BG hybrid molecules. Reagents and conditions: (**a**) *N*-Bromosuccinimide (1.0 eq.), dibenzoyl peroxide (2 mol-%), CCl_4_, reflux, 20 h. (**b**) NaN_3_ (3.0 eq.), DMF, room temp., 5 h. (**c**) PPh_3_ (2.1 eq.), acetone, room temp., 1.5 h; then H_2_O (26 eq.), NEt_3_ (1.6 eq.), 2 (1.4 eq.), room temp., 2 d. (**d**) 10 (6.0 eq.), CuI (1.0 eq.), Cs_2_CO_3_ (2.0 eq.), isopropanol, room temp., 4 d. (**e**) PPh_3_ (1.1 eq.), hexachloroethane (1.1 eq.), CH_2_Cl_2_, room temp., 20 h. (**f**) F_3_CCOOH/CH_2_Cl_2_ 1:1, room temp., 30 min.

Our syntheses of the **MBG** hybrids mM*BG and pM*BG—which we isolated as the corresponding trifluoroacetates mM*BG·TFA and pM*BG·TFA—started from the commercially available iodotoluenes *meta*- and *para*-**6**, respectively (Scheme 2). Solutions of these compounds in CCl_4_ were subjected to Wohl-Ziegler brominations. They rendered the corresponding benzyl bromides *meta*-**7** (69% yield) and *para*-**7** (57% yield), respectively. Exposure to solutions of sodium azide in DMF engaged these compounds in S_N_2 reactions, which afforded the azides *meta*- and *para*-**8** yields of 58% and 80%, respectively. Each of these compounds is susceptible to a tandem transformation [26] beginning with a Staudinger reduction (PPh_3_; H_2_O) and ending with an amidinylation with the *N*,*N*’-di-Boc-protected thiourea **2** [23]. This furnished the *N*,*N*’-di-Boc-protected benzylguanidines [24] *meta*-**9** (= Boc_2_**mIBG**; 57%) and *para*-**9** (= Boc_2_**mIBG**; 66%). Like ordinary aryl iodides, the last-mentioned compounds and aminoethanol underwent Ullmann-type monoaminations/monoarylations [27]. They rendered the (HO-CH_2_-CH_2_-)NH-containing *N*,*N*’-di-Boc-protected **BG**s [24] *meta*-**11** (64% yield) and *para*-**11** (62% yield), respectively. The OH group of these compounds was converted into Cl by treatment with hexachloroethane and PPh_3_ [28]. This delivered the (HO-CH_2_-CH_2_-)NH-containing *N*,*N*’-di-Boc-protected BGs [27] *meta*-**12** (59% yield) and *para*-**12** (53% yield). Treatment of these compounds with TFA/CH_2_Cl_2_ (1:1, *v*:*v*) removed the Boc groups and delivered, after evaporation of all volatiles, the (Cl-CH_2_-CH_2_-)NH-containing benzylguanidine trifluoroacetates mM*BG·TFA and pM*BG·TFA in quantitative yields. Overall, their syntheses comprised 6 steps in the longest linear sequence and succeeded in total yields of 7% and 11%, respectively.

Remarks to Scheme 1 and Scheme 2, Ref. [24]: All[MP1] *N*,*N*’-di-BOC[M2]-protected guaninides encountered in the present study, i.e., the *meta* and *para* isomers of compounds **3**, **5**, **9**, **11**, and **12**, represented one single of two conceivable tautomers. The ^1^H-NMR shifts of the two N-bound protons within each of these compounds differed from one another grossly. This let us conclude that these protons are 1 ξ CH_2_-NH (8.43 ppm ≤ δ_CH_2_-N*H* in CDCl_3_ solution_ ≤ 8.65 ppm) plus 1 ξ NHBoc (δ_N*H*-Boc in CDCl_3_ solution_ = 11.51 or 11.52 ppm), not 2 × NHBoc.

### 2.2. Cell Culture and Cell Culture Experiments

#### 2.2.1. Human Neuroblastoma Cell Lines

Human Neuroblastoma Cell Lines:

(a) SK-N-SH (American Type Culture Collection, ATCC; MD, USA), Nr. HTB-11 [29];
(b) Kelly (German Collection of Microorganisms and Cell Cultures, DSMZ, Braunschweig, Germany), ACC 355 [30];
(c) LS (DSMZ, ACC 675); (established in our laboratory) [31].

Cells were grown as monolayers in RPMI 1640 medium supplemented with 10% inactivated FCS, L- glutamine (2 mmol/L), and penicillin/streptomycin (100 U/mL; 100 µg/mL) in 250 mL cell culture flasks at 37 °C, 5% CO_2_ and 100% humidity. For cell experiments, cells were detached by trypsin, washed with PBS^++^, and resuspended in the respective incubation medium.

#### 2.2.2. Inhibition of [^3^H]noradrenaline Uptake in Neuroblastoma Cells: Competitive Studies with MBG Hybrid Molecules Compared to mIBG and M

To examine the uptake into the human neuroblastoma cell lines SK-N-SH (high NAT expression), Kelly (moderate NAT expression), and LS (very low NAT expression), competitive uptake experiments with [^3^H]noradrenaline {[^3^H]NA} were performed {noradrenaline (Norepinephrine), LL evo-[7-^3^H]; specific activity: 10-30 Ci (370 GBq-1.11 TBq/mmol); concentration 1mCi/mL; PerkinElmer, Freiburg, Germany}. [^3^H]noradrenaline is equally taken up by the NAT as radio-labelled **mIBG**. The competitive uptake studies were carried out in analogy to the experiments described previously with busulfan benzylguanidine (**BBG**) hybrids [21]. Briefly, the adherent growing neuroblastoma cell lines were detached with trypsin and, after a short regeneration period, used as suspension culture (usually 500 µL; 1 × 10^5^ cells/mL phosphate buffered saline + Ca^++^/Mg^++^ (PBS^++^) + glucose (5.5 mmol/L)). In addition, 500 µL cell suspensions were incubated with 10 µL noradrenaline (final concentration: 0.1 µmol/L) spiked with [^3^H]NA for 15 min at 37 °C. After centrifugation, cell pellets were lysed with 500 µL distilled water and mixed with 10 mL of liquid scintillation cocktail (OptiPhase Super Mix, PerkinElmer). The amount of NA (fmoles/10^6^ cells) taken up by cells in controls was calculated by measurement of radioactivity taken up by cells and set as 100%. The uptake of [^3^H]noradrenaline in the presence of an excess of unlabelled **mIBG**, M, mM*BG, pM*BG, **mMBG**, and **pMBG** was given as % uptake compared to PBS^++^ controls.

#### 2.2.3. MTS-Test (Viability Test)

Substances were tested in the concentration range from 0.01 µmol/L to 1000 µmol/L using the CellTiter 96^®^ AQ_ueous_ One Solution Cell Proliferation Assay (MTS) from Promega (Walldorf, Germany). MTS [3-(4,5-dimethylthiazol-2-yl)-5-(3-carboxymethoxyphenyl)-2-(4-sulphophenyl)-2H-tetrazolium, inner salt in combination with a reductant (phenazine methosulfate) was used. The test is a colorimetric assay for determining the viable cells by bioreduction to a formazan product. The influence of the respective substances was measured on cell viability. The assay was performed according to manufacturer’s instruction in 96 flat bottom cell culture plates. In addition, 200 µL of cell suspension (SK-N-SH: 2.5 × 10^4^ cells/well; Kelly: 1.5 × 10^4^ cells/well; LS: 1 × 10^4^ cells/well) in cell culture medium was transferred to the wells of a 96-well plate. After 24 h, 10 µL of the respective substances and concentrations was added in triplicates to the wells. The incubation was stopped after 72 h of incubation at 37 °C/5% CO_2_ and the samples were analysed according to the test protocol. The viability of the cells after treatment with the respective substance concentrations was related to the control samples (incubation in cell culture medium), set as 100% cell viability. Each experiment was carried out in three independent runs.

#### 2.2.4. CELLigence Assay

In order to gain detailed information concerning the effectivity of the hybrid molecules during the proliferation of the neuroblastoma cells, real-time neuroblastoma cancer cell proliferation was determined using the xCELLigence SP system (Roche Applied Science, Mannheim, Germany). In contrast to the MTS assay, the xCELLigence assay allowed us to follow the growth of cells continuously in the absence and presence of the added drugs.

Neuroblastoma cells were seeded in 96-well plates (E-Plate 96, ACEA Biosciences, San Diego, CA, USA), 20,000 cells/well. Real-time dynamic cell proliferation was monitored in 30 min intervals for 96 h at 37 °C. 24 h after seeding, and cells were treated with **M** and the **MBG** hybrids, as indicated. Cell index values were calculated using the RTCA Software (2.0). All curves were normalised to the time point after drug treatment was conducted (~24 h after seeding) applying the RTCA software [32,33].

## 3. Results

### 3.1. Synthesis of Our Four MBG Hybrid Compounds

The syntheses of the four hybrid compounds are described in detail in Section 2.1. The syntheses of **mMBG**·TFA and **pMBG**·TFA comprised 3 steps in the longest linear sequence and succeeded in total yields of 38% and 27%, respectively. The syntheses of mM*BG·TFA and pM*BG·TFA comprised 6 steps in the longest linear sequence and succeeded in total yields of 7% and 11%, respectively.

### 3.2. Stability of the Newly Synthesised MBG Hybrid Compounds

Once any of our **MBG** hybrids, i.e., **mMBG**, **pMBG**, mM*BG, and pM*BG, had been synthesised as the respective trifluoroacetate **mMBG**·TFA, **pMBG**·TFA, mM*BG·TFA, or pM*BG·TFA, they were stored in the freezer at −20 °C. Trifluoroacetate (TFA) was chosen as a counter ion for each respective benzylguanidinium moiety because the corresponding salts were highly soluble in aqueous media. Storage at this temperature was possible for several to many weeks, neither affecting the integrity nor the homogeneity of the respective compound. In order to estimate these compounds’ stabilities under the conditions of our cell culture experiments (see below), an aqueous solution of the compound of interest was incubated at 37 °C. After 1 h or 5 h of such exposure, ^1^H-NMR spectroscopy (300.1 MHz) in DMSO-d_6_ solution revealed to which extent the respective **MBG** hybrid had survived the heat stress (Table 1). The least stable compound was the dialkylating agent **mMBG**·TFA, whereas the most stable compounds were the monoalkylating agents mM*BG·TFA and pM*BG·TFA.

### 3.3. Interaction of Our MBG Hybrid Compounds with Neuroblastoma Cell Lines with Different NAT Expressions

#### 3.3.1. Inhibition of [^3^H]noradrenaline Uptake in Neuroblastoma Cells: Competitive Studies with MBG Hybrid Molecules Compared to mIBG and M

**Figure 2:**
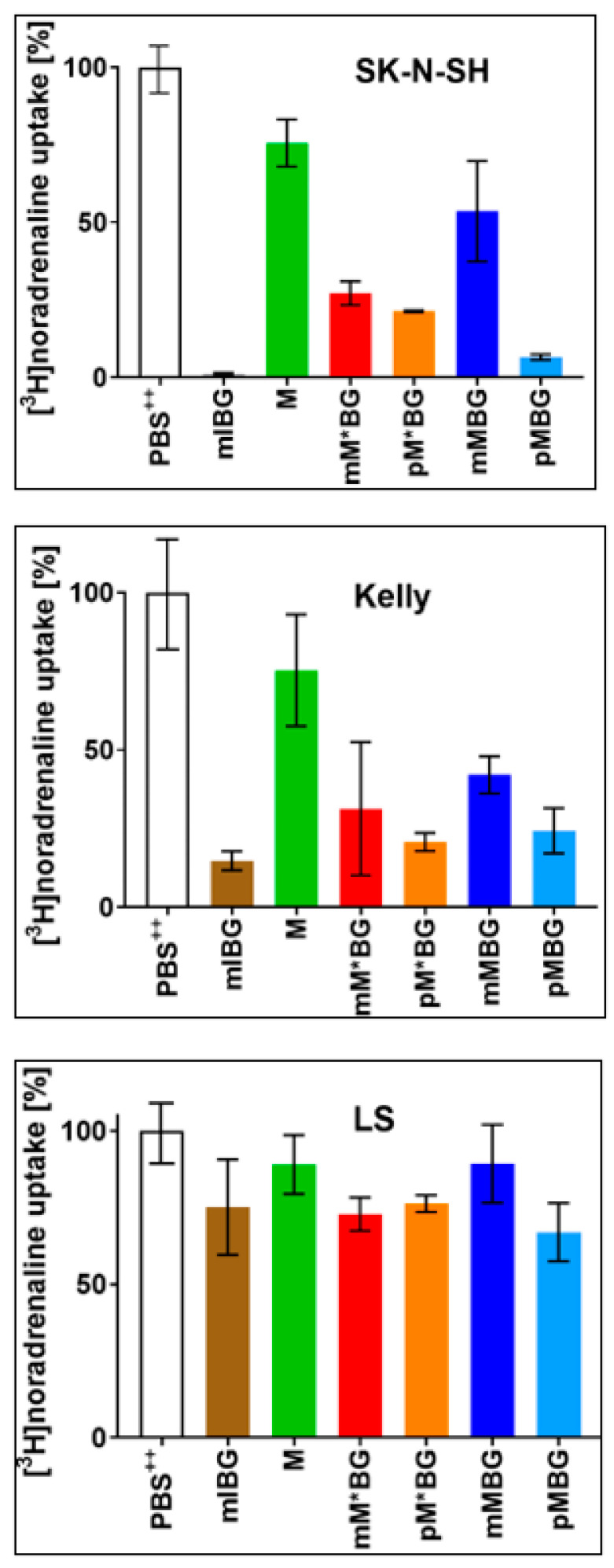
Uptake of [^3^H]noradrenaline (*c* = 0.1 µmol/L) within 15 min at 37 °C into neuroblastoma cells with different expressions of the NAT (SK-N-SH > Kelly >> LS). This uptake was measured in a PBS^++^ (+5.5 mmol/L glucose) control, designated as “100% uptake” and in the presence of a molar excess of one of the following potential competitors (*c* = 100 µmol/L): **mIBG**·HCl (■), **M** (■), mM*BG·TFA (■), pM*BG·TFA (■), **mMBG**·TFA (■), and **pMBG**·TFA (■). Mean value ± S.D based on 3 independent sets of experiments, each of which was performed in triplicate.

Competitive uptake experiments of [^3^H]noradrenaline {[^3^H]NA} in the presence of **mIBG**, **M**, and the **MBG** hybrids were performed with neuroblastoma cell lines with high NAT expression (SK-N-SH), moderate NAT expression (Kelly), and no NAT expression (LS) (for method, see Section 2.2.2). As expected, **mIBG** is the most potent inhibitor of [^3^H]NA uptake via the NAT [34]. Figure 2 shows that the hybrids—in contrast to **M**—significantly inhibited the uptake of [^3^H]NA in the NAT-expressing neuroblastoma cell lines SK-N-SH and Kelly, but not LS cells.

#### 3.3.2. Viability Analyses (MTS Assays) of MBG Hybrid Compounds

**Figure 3:**
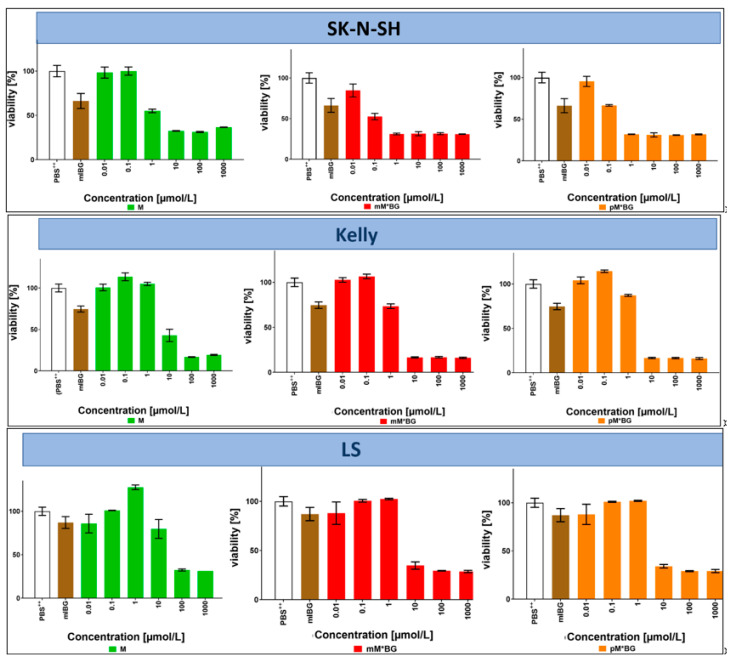
MTS viability assays with the neuroblastoma cell lines SK-N-SH, Kelly, and LS after incubations with the indicated compounds at 37 °C for 72 h: Effects of **M** (left: **■**), mM*BG·TFA (centre: **■**) and pM*BG·TFA (right; **■**) administered in concentrations of 0.01, 0.1, 1, 10, 100, and 1000 µmol/L (corresponding to logarithmic horizontal scales). The extreme left of each diagram depicts the corresponding PBS^++^ (+ 5.5 mmol/L glucose) control (whose MTS assay served to define “100% viability”) and a reference treatment with **mIBG**·HCl (■), administered only in a concentration of 10 µmol/L. Mean ± S.D; 3 independent experiments, each in triplicate.

**Figure 4:**
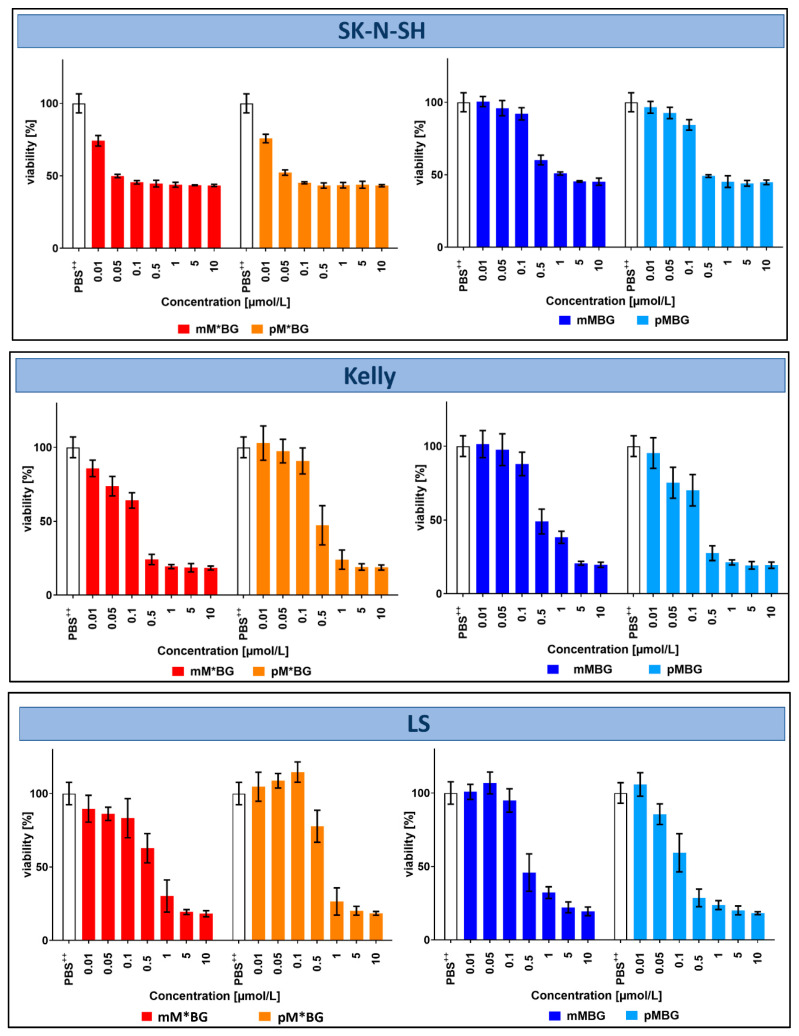
MTS viability assays with the neuroblastoma cell lines SK-N-SH, Kelly, and LS after incubations with the indicated compounds at 37 °C for 72 h: Effects of mM*BG·TFA (**■**) and pM*BG·TFA (**■**) (left), compared to **mMBG**·TFA (**■**) and **pMBG**· TFA(**■**) (right), administered in concentrations of 0.01–0.05–0.1–0.5–1–5–10 µmol/L (corresponding to near logarithmic horizontal scale). The extreme left of each diagram depicts the corresponding PBS^++^ (+ 5.5 mmol/L glucose) control (whose MTS assay served to define “100% viability”). Mean ± S.D; 3 independent experiments, each in triplicate.

Cell viability assays measure the reduction of MTS by NADPH and/or NADH, which are generated by dehydrogenases in metabolically active cells. Potentially toxic drugs can lead to impaired activity, associated with reduced cell proliferation and finally to cell death. In the experiments shown in Figure 3, the effects of monoalkylating **MBG**s (mM*BG and pM*BG) on cell viability were compared with the effects of **mIBG** and **M** (dialkylating **MBG**s were not obtainable at this time). In later experiments shown in Figure 4, the effects of dialkylating **MBG**s (**mMBG** and **pMBG**) were directly compared to the effects of monoalkylating **MBG**s (mM*BG and pM*BG).

After an incubation period of 72 h, clear effects were observed from about 10 µmol/L, in SK-N-H cells already at 1 µmol/L. Both M*BGs were more effective than **M** and **mIBG**·HCl, the isomers mM*BG·TFA and pM*BG·TFA being similarly effective.

Taken together, the effects of **BG** hybrids with only one alkylating group (mM*BG/pM*BG) were similar or even a little bit better than those with two alkylating groups (**mMBG**/**pMBG**).

#### 3.3.3. Proliferation Kinetics of Neuroblastoma Cells (xCELLigence Assay) of MBG Hybrid Compounds

In order to gain detailed information concerning the effectivity of the hybrid molecules during the proliferation of the neuroblastoma cells, real-time neuroblastoma cancer cell proliferation was determined using the xCELLigence SP system (Roche Applied Science, Mannheim, Germany). In contrast to the MTS assay, the xCELLigence assays allowed us to follow the growth of cells continuously in the absence and presence of the added drugs.

**Figure 5:**
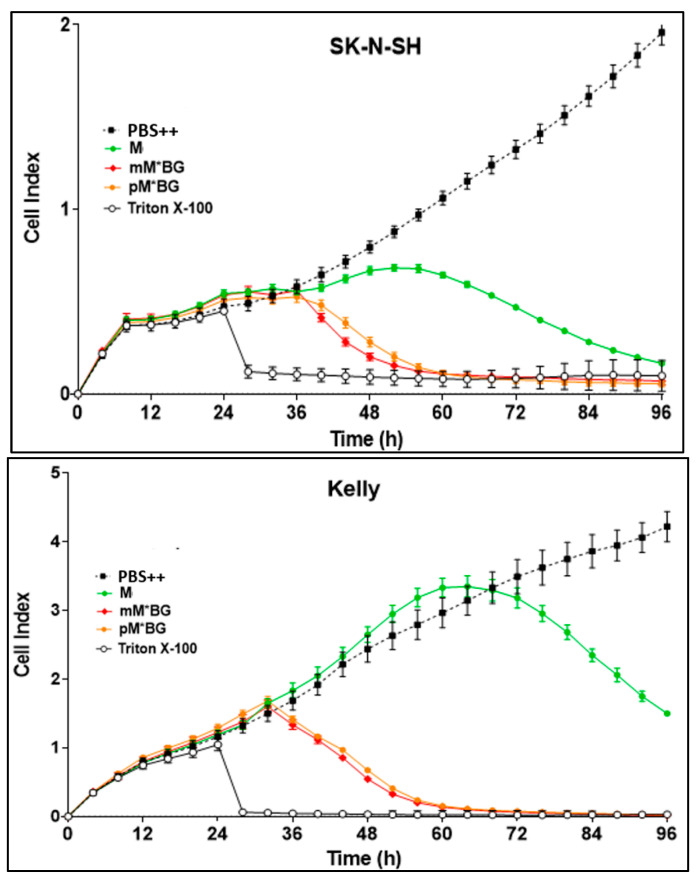

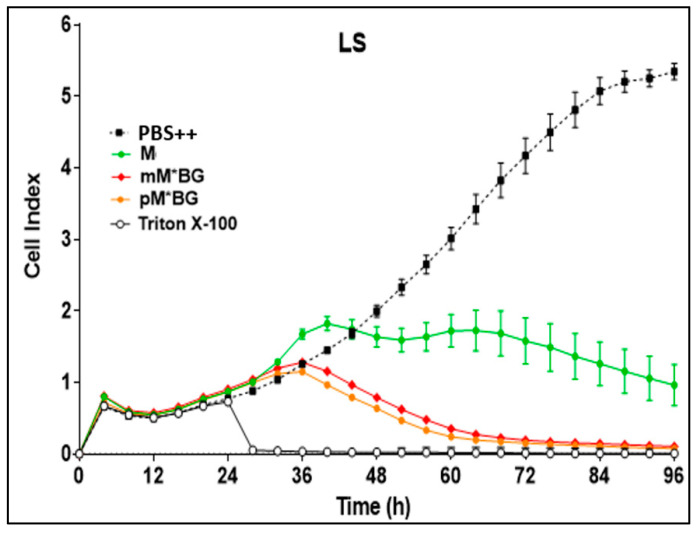
Real-time proliferation monitoring over 96 h in the presence of **M** (green line), mM*BG·TFA (red line), and pM*BG·TFA (orange line) compared to controls (PBS ^++^ + 5.5 mmol/L glucose, black line). Following the initial tumour cell seeding (at hour 0), the neuroblastoma cells SK-N-SH, Kelly, and LS were treated 24 h later with 10 µmol/L (final concentration) of **M** and the M*BG hybrids. Treatment with Triton X-100 1%, inducing maximum tumour cell lysis, was used as a negative control. The cell index was normalised after 24 h when treatment had been accomplished. Mean ± S.D.; 3 independent experiments each in triplicate.

**Figure 6:**
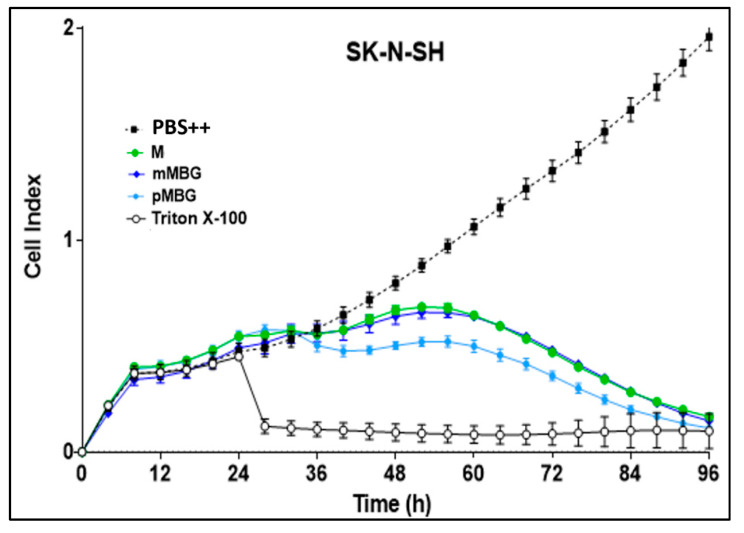

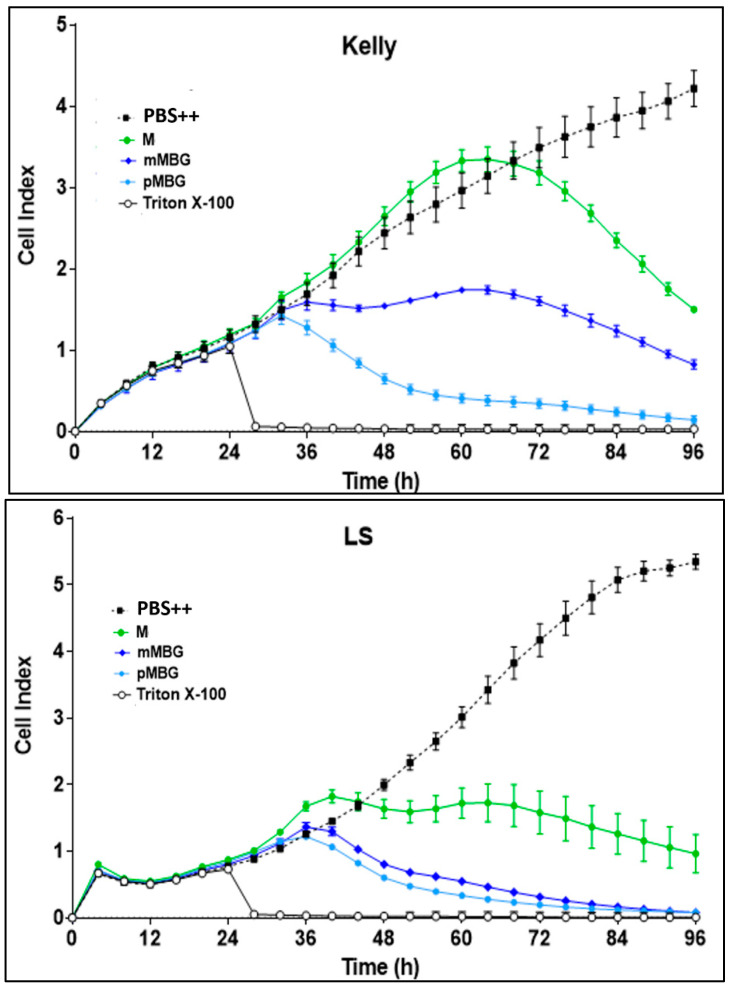
Real-time proliferation monitoring over 96 h in the presence of **M** (green line), **mMBG**·TFA (dark blue line), and **pMBG**·TFA (light blue line) compared to controls (PBS ^++^ + 5.5 mmol/L glucose, black line). Following the initial tumour cell seeding (at hour 0), the neuroblastoma cells SK-N-SH, Kelly, and LS were treated 24 h later with 10 µmol/L (final concentration) of **M** and the **MBG** hybrids. Treatment with Triton X-100 1%, inducing maximum tumour cell lysis, was used as a negative control. The cell index was normalised after 24 h when treatment had been accomplished. Mean ± S.D.; 3 independent experiments each in triplicate.

In screening experiments, the effects of **M** and the **MBG**s were first investigated in the concentration range of 0.1–100 µmol/L in order to estimate acceptable concentrations for further experiments: 1–10 µmol/L (final concentration in the cell cultures) proved to be well suitable. The following experiments shown in Figure 5 and Figure 6 were carried out using a drug concentration of 10 µmol/L.

Figure 5 shows that the hybrids with one alkylating group (mM*BG·TFA and pM*BG·TFA) proved to be more effective than **M** to all three cell lines.

The hybrids with two alkylating groups (**mMBG** TFA and **pMBG**·TFA) were more toxic than **M** in Kelly and LS cells, but less pronounced in SK-N-SH cells.

## 4. Discussion

To an important extent, the chemotherapy of cancer relies on the in vivo alkylation of biomolecules by alkylating agents [35,36]. The earliest used examples were the compounds (Cl-CH_2_-CH_2_-)_3_N and (Cl-CH_2_-CH_2_-)_2_N-Me [37,38,39]. The last-mentioned compound is the simplest representative of a class of compounds containing the structural motif (Cl-CH_2_-CH_2_-)_2_N-. They possess high alkylating power, which makes them akin to, and possibly even superior to, the alkylating agent (Cl-CH_2_-CH_2_-)_2_S. The latter was dubbed “mustard gas” during WWI warfare. This is why compounds containing the motif (Cl-CH_2_-CH_2_-)_2_N- are commonly referred to as “N-mustards”. N-mustards—not only **M** [14]—played a pivotal role in the development of chemotherapy for cancer [40,41,42,43,44,45]. Their development is continuing at a considerable pace [46,47,48,49,50,51]. The present study contributes its share to this field.

As already described, the alkylating chemotherapeutics busulfan (**B**) and melphalan (**M**) have been successfully introduced into the therapy of high-risk neuroblastoma before stem cell transplantation [16]. Due to their toxicity, especially on hematopoietic stem cells, both substances are not used in conventional chemotherapy of neuroblastoma without stem cell transplantation. Therefore, we decided to synthesise hybrid molecules of benzylguanidine (with the aim of more specific uptake into neuroblastoma cells) and the alkylating groups of these cytostatic drugs **B** and **M**. In a preceding paper, we reported the effects of **mBBG** and **pBBG** (busulfan benzylguanidine hybrids) compared to **B** {busulfan, 5-[(methanesulfonyl)oxy]pentyl methanesulfonate} [21]. In the actual manuscript, we present our results concerning the effects of **MBGs** (melphalan benzylguanidine hybrids) on neuroblastoma cells.

As already mentioned in the introduction, it is obvious that the alkylating cytostatic drug **M** has a fundamental handicap: the carrier structure of its alkylating group is **Phe**, which can probably be taken up by almost all cell types by amino acid transporters [17,18]. This is probably the reason for the unspecific side effects of **M**. Nevertheless, **M** proved to be very effective in the conditioning regime in high-risk neuroblastoma for the destruction of neuroblastoma cells and hematopoietic cells before transplantation [16,52]. In order to reduce the side effects observed during treatment with **M**, our aim was to synthesise hybrid molecules consisting of the high effective alkylating group of **M** and a carrier group (benzylguanidine), which can be taken up by the NAT of neuroblastoma cells. However, their uptake is not limited to neuroblastoma cells: **mIBG** (and probably the **MBGs**) can also be incorporated by cells expressing organic cation transporters (OCTs), such as kidney and liver cells, but uptake via these transporters can be reduced by clinically approved corticosteroids [53]. Therefore, a combination of corticosteroids with [^123^I]**mIBG**/[^131^I]**mIBG** or with **MBGs** should enhance their preferred uptake via the NAT and thereby improve diagnosis and therapy of neuroblastoma.

For synthesis, the alkylating group (Cl-CH_2_-CH_2_-)_2_N- of **M** binds to benzylguanidine in the *meta*- (instead of iodine in mIBG) and *para*-position. Furthermore, hybrids with only one alkylating group, (Cl-CH_2_-CH_2_-)NH-, bound in the *meta*- and *para*-position, were also synthesised. All four **M** hybrids were at least equally as effective, but in most cases more effective, on neuroblastoma cell cultures than **M**. Surprisingly, hybrids containing only one alkylating group were at least equally as effective as those with two alkylating groups. In **M**, the two activated alkylating, positively charged groups can crosslink the strands of DNA by reacting with the nucleophilic amino groups comprising guanine N-7 and adenine N-3 [54,55,56]. Concerning the **M** hybrids mM*BG and pM*BG with only one alkylating group, they are definitely unable to crosslink DNA. However, their alkylating power towards the mentioned nucleophilic amino groups containing guanine N-7 and adenine N-3 should be essentially the same as the monoalkylation power of their bifunctional analogues **mMBG** and **pMBG**. This allows us to speculate that the more pronounced effects of the hybrids mM*BG and pM*BG with only one alkylating group sometimes observed may be, at least partly, associated their higher thermal stability (Table 1).

Comparing the three neuroblastoma cell lines with different NAT expression, it was conspicuous that LS cells (very low NAT expression) were similarly sensitive against the **M** hybrids than SK-N-SH and Kelly cells. This aspect needs to be investigated in more detail with other cells lacking NAT expression. We suppose that the presence of OCTs in LS cells is responsible for the uptake of the **M** hybrids (not yet investigated).

## 5. Conclusions

Our newly synthesised **M** hybrids **mMBG** and **pMBG** containing the alkylating entity (Cl-CH_2_-CH_2_-)_2_N- of **M**—two 2-chloroethyl groups bound to one N—proved to be more effective against neuroblastoma cell lines as the equally (Cl-CH_2_-CH_2_-)_2_N-containing drug **M**, indicating their potential as an improved chemotherapeutic drug in neuroblastoma therapy. Although our original intention was to only synthesise molecules with strict homologies to **M**, we found that the less closely related, **M**-inspired (Cl-CH_2_-CH_2_-)NH-containing benzylguanidine hybrids mM*BG and pM*BG—with only one 2-chloroethyl group bound to the N atom—were also effective in neuroblastoma cells. This may be due to—either altogether or in part—mM*BG and pM*BG being more thermally stable than **mMBG** and **pMBG** during incubation at 37 °C.

All four hybrid molecules were at least equally as cytotoxic, but in most cases even more cytotoxic against the neuroblastoma cells than melphalan in vitro. Based on these data, we conclude that all four hybrids should essentially be suitable for neuroblastoma therapy. However, further experiments in vitro and in vivo are necessary in order to support this concept. Next, in vitro experiments will be done with an expanded set of well characterised, commercially available neuroblastoma cell lines together with non-neuroblastoma cells (kidney, liver, fibroblast, and hematopoietic stem cells) in order to comparatively analyse the cytotoxicity and uptake of the MBGs and melphalan by the noradrenaline transporter, amino acid transporter, and organic cation transporter in detail. The influence of glucocorticoides for a more selective uptake via the noradrenaline transporter will also be analysed. Subsequently, mouse experiments (the toxicity and treatment of the neuroblastoma-bearing mouse) should be performed with melphalan and the most suitable MBGs.

## Figures and Tables

**Table 1:** Fraction of originally intact **MBG** hybrid molecules (formulas: Figure 1) remaining unaltered after having been maintained at +37 °C in aqueous solutions ^a^.

Compound	Stability After 1 h of Incubation at +37 °C	Stability After 5 h of Incubation at +37 °C
**mMBG**·TFA	79%	unstable
**pMBG**·TFA	87%	70%
mM*BG·TFA	>98%	92%
pM*BG·TFA	>98%	92%

^a^ After incubation, the respective solution was frozen. The water was removed by freeze-drying. The remaining solid was dissolved in DMSO-d_6_. The resulting solution was analysed by ^1^H-NMR spectroscopy (300.13 MHz).

## Data Availability

(a) Synthesis of the MBG hybrids: Lischka, M. Synthese cytotoxischer Hybridmoleküle aus Benzylguanidin und Alkylantien zur Therapie Kupplung: Synthese 5.5′-verbrückter 1,1′-Biphenyl-2,2′-diphosphane und deren Einsatz in katalytisch-asymmetrischen 1,4-Additionen von Arylboronsäuren an cyclische Enone. Doctoral Thesis, Albert-Ludwigs-Universität Freiburg, Freiburg im Breisgau, Germany, 2019. (https://www.zvab.com/Synthese-cytotoxischer-Hybridmolekule-Benzylguanidin-Alkylantien-Therapie/31007703557/bd accessed on 11 May 2023). (b)Experiments using three neuroblastoma cell lines, obtained from: SK-N-SH (American Type Culture Collection, ATCC; MD, USA), Nr. HTB-11; Kelly (German Collection of Microorganisms and Cell Cultures, DSMZ, Braunschweig, Germany), ACC 355; LS (DSMZ, ACC 675).
